# Trapeziometacarpal Joint Arthroplasty of the Thumb without Osseous Tunnels and Carpal Tunnel Release via a Radial Approach; Technique, and Results

**DOI:** 10.1055/s-0039-1697635

**Published:** 2019-09-24

**Authors:** Chung Ming Chan, Efraín Farías Cisneros, Tsu-Min Tsai

**Affiliations:** 1 Department of Orthopaedics and Rehabilitation, University of Florida, Gainesville, Florida; 2 Department of Orthopedics, Hospital Español de México, Mexico City, Mexico; 3 Christine M. Kleinert Institute for Hand and Microsurgery, Louisville, Kentucky

**Keywords:** arthritis, arthroplasty, carpometacarpal joint, thumb, trapeziectomy

## Abstract

**Background** Numerous surgeries have been described for osteoarthritis of the trapeziometacarpal (TMC) joint. We describe the senior author's experience with his technique of concurrent arthroplasty of the TMC joint, and carpal tunnel release (CTR) via a radial approach.

**Methods** The study is a case series of patients managed over a 3-year period. We included 86 patients over 40 years of age that had concurrent CTR. We used the paired *t* -test to compare the preoperative and postoperative grip strength and functional scoring (including the Levine-Katz questionnaire for carpal tunnel syndrome, disabilities of the arm shoulder and hand [DASH] score and QuickDASH9).

**Results** Mean age at surgery was 62.8 years, and mean follow-up was 13.1 months. Functional outcomes were analyzed in 65 patients. Grip strength returned to the preoperative measurement by 3 months. Analysis of the nine patients followed up for more than 13 months postoperatively showed a significant increase in grip strength at last follow-up. The grip strength in both hands was also similar beyond 13 months. Significant decreases in the functional scores recorded indicated a reduction in disability, symptom severity, and functional impairment.

**Conclusions** In conclusion, we present the favorable results of this technique of TMC arthroplasty and CTR involving no bone tunnels and short-term immobilization.

Osteoarthritis (OA) of the trapeziometacarpal (TMC) joint at the base of the thumb is a common condition that causes considerable disability. 1 2 Surgery is indicated for symptomatic relief of moderate-to-severe disease and numerous surgical procedures have been described for this. Most of these procedures involve either a complete or partial trapeziectomy (the distal articular portion), and they differ in whether or not a ligament reconstruction is performed. 3 4 5 6 The techniques also differ in the use of bone tunnels and the choice of tendon used in tendon interposition or reconstruction when performed. Well-known techniques include ligament reconstruction and tendon interposition utilizing a portion of the flexor carpi radialis (FCR) tendon popularized by Burton and Pellegrini, 4 and the suspensionplasty technique using half of the abductor pollicis longus (APL) tendon described by Thompson. 6 Systematic reviews have not been able to find superiority of any particular mode of surgical treatment over another. 7 8

In patients with TMC arthritis undergoing arthroplasty, carpal tunnel syndrome (CTS) frequently is present, with estimates ranging for 16.7 9 to 43%. 10 Carpal tunnel release to address CTS is commonly performed via an incision between the thenar and hypothenar eminences, or with the use of an endoscope along this same anatomical path. The radial approach to the carpal tunnel release (CTR) was described by Weber and Sanders 11 with the notable advantages of decreased disruption of glabrous palmar skin and soft tissue, and positioning of the median nerve away from the scar. Considering the relatively high incidence of CTS and TMC OA, performing carpal tunnel release routinely with TMC joint arthroplasty has been advocated, 10 and this is the practice of the senior author when managing patients with TMC OA.

The senior author has been using the technique described below for the past 15 years when managing patients with TMC OA surgically. This study describes the author's experience with his technique and postoperative protocol.

## Methods

The study is a retrospective cohort study of patients managed by the senior author. Approval for the study was obtained from the institutional review board for this study. The senior author's surgical database was queried to identify all patients who had undergone trapezial resection and arthroplasty of the carpometacarpal joint of the thumb for degenerative joint disease of the TMC joint from 2011 to 2013. Patients included in the study were (1) over the age of 40 years at the time of surgery, (2) were diagnosed with CTS clinically from history and on examination findings (e.g., positive Tinel's sign, Phalen's test, and/or Durkan's test), (3) underwent this surgery for Eaton and Littler's stage II to IV arthritis, and (4) had radial approach to release the carpal tunnel concurrently. Patients were excluded if they had (1) surgery for inflammatory arthritis, (2) concurrent surgery on the carpal bones or distal radius that required modification of the postoperative rehabilitation protocol, and (3) required modification of the technique owing to a ruptured FCR tendon

The standard postoperative follow-up protocol included visits postoperatively at 2 weeks, 6 weeks, 3 months, and thereafter at 6 months and 1 year. We logged any measurements of function obtained during preoperative and postoperative clinic visit as documented on the clinical charts. These included grip strength dynamometry (using JAMAR) and functional scores, including the Levine-Katz 12 (Boston) questionnaire for CTS, disabilities of the arm shoulder and hand (DASH) score, and the QuickDASH9 to assess the extent of disability from upper extremity conditions. These questionnaires, where administered, were submitted from the preoperative visit and at the 3 months follow-up visit.

Statistical analysis was conducted using the statistics program EZR 13 a statistical software package based on R (version 2.13.0). Characteristics of the study population were characterized by descriptive statistics, while comparison of preoperative and postoperative parameters was performed with the use of the paired *t* -test.

During the study period, 114 patients underwent the surgery described for stage II to IV degenerative joint disease. Of these 114 patients, 14 did not undergo concurrent carpal tunnel release, 8 underwent concurrent wrist surgery that required modification of the postoperative rehabilitation protocol, and 6 required modification of the reconstructive technique owing to a frayed or ruptured FCR tendon. Eighty-six patients met study criteria and of these 86, preoperative and postoperative grip strength dynamometry scores were available for analysis of 65 patients, and complete records of preoperative and postoperative functional scoring were available for 23 patients.

### Description of Technique

The skin incision comprises a curvilinear Wagner incision along the border of glabrous and nonglabrous skin extending from the level of the mid thumb metacarpal extending proximally to the scaphoid tubercle, and a “z” shaped proximal extension over the distal end of the FCR tendon. Elevation of the thenar musculature exposes the TMC and scaphotrapezial joint and the first metacarpal base. The FCR tendon sheath is opened and the bony crest of the trapezium overlying the FCR is removed with a rongeur and the tendon is then displaced radially.

The carpal tunnel release is then performed as described by Tsai et al. 14 The deep portion of the TCL (dTCL), which comprises the floor of the FCR tendon sheath, is incised longitudinally resulting in the release of the carpal tunnel. The flexor pollicis longus (FPL) tendon is well visualized at this point. Another incision is made parallel to the first incision of the dTCL creating a distally based ligamentous flap that is then sutured back to the radial sided leaflet of the dTCL to create a pulley to mitigate bowstringing of the FPL tendon.

**Fig. 1:**
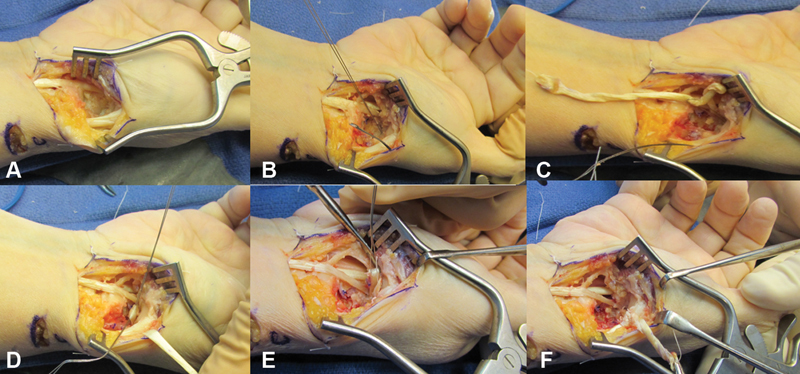
Operative photographs. ( **A** ) View of space created following trapeziectomy. ( **B** ) Following placement of wire facilitating passage of flexor carpi radialis (FCR) graft dorsal to base of thumb metacarpal. ( **C** ) FCR graft being passed dorsally. ( **D** ) Wire loop placed in loop of FCR graft to facilitate passage knotting of FCR graft. ( **E** ) Wire loop used to pull end of the FCR graft through the loop of FCR in the intermetacarpal space. ( **F** ) FCR being tied to itself at the abductor pollicis longus insertion creating a suspensory sling.

The total trapeziectomy is then performed with a combination of osteotomes, rongeur, and sharp dissection. A partial trapezoidectomy is also performed to prevent impingement of the base of the thumb metacarpal with the trapezoid, as ∼5mm proximal migration of the thumb metacarpal is anticipated. This is performed by removing the portion of the trapezoid that protrudes radial to a line extending proximally from the ulnar border of the thumb metacarpal ( Fig. 1A ). Attention is then turned to the ligament reconstruction with a distally based FCR tendon graft. A volar transverse incision is made over the region of the musculotendinous junction of the FCR, and ∼40% of the width of the FCR is harvested. The senior author's preferred technique for harvesting the FCR is the antegrade passage of a tendon stripper, and dissection of the distal portion of the FCR tendon under direct vision. Handling and fraying of the tendon end are minimized by passing a short segment of suture through the proximal end of the tendon graft to allow grasping of suture for graft manipulation instead of direct grasping of the tendon end.

**Fig. 2:**
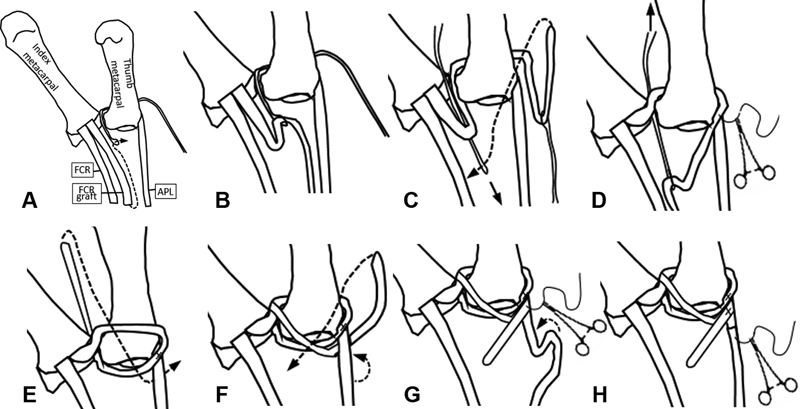
Schematic representation of steps of ligament reconstruction from a volar projection depicting the use of wire loops to weave the distally based slip of flexor carpi radialis (FCR) tendon starting from the base of the index metacarpal, dorsal to the base of the thumb metacarpal to the abductor pollicis longus (APL) insertion, then back to the FCR insertion at the index metacarpal base, and then back to the APL insertion again.

This distally based FCR tendon graft is then woven with the help of a right-angled hemostatic forceps and a wire loop to create the ligament reconstruction to support to metacarpal base. The right-angled hemostatic forceps are first inserted from the radial side of the base of the thumb metacarpal in between the APL and extensor pollicis brevis (EPB) tendons just distal to the APL insertion. The tips of the forceps are guided around the dorsal surface of the thumb metacarpal and made to exit into the cavity created by the trapeziectomy on the ulnar side of the base of the thumb metacarpal; the tips emerge between the bases of the thumb and index metacarpals just proximal to the intermetacarpal ligament. The hemostatic forceps are then replaced by a wire loop by grasping the terminal ends of the wire loop and retrieving the hemostatic forceps by reversing its path of introduction ( Fig. 1B , Fig. 2A ).

The FCR tendon graft is then routed dorsal to the thumb metacarpal base by placing the tendon in the wire loop and retrieving the loop from the volar–radial side of the thumb ( Fig. 1C , Fig. 2B ). Prior to the FCR tendon graft being completely pulled through and disappearing from sight within the trapeziectomy cavity, another wire loop is placed from distal to proximal through the loop of FCR graft that is being pulled through ( Fig. 1D , Fig. 2C ).

Traction is applied to the thumb and the FCR tendon graft is sutured to the APL just proximal to its insertion, as it emerges between the APL and EPB. The wire loop placed dorsal to the FCR tendon graft in the earlier step is then used to pass the free end of the tendon graft from proximal to distal by retrieving the wire loop distally ( Fig. 1E , Fig. 2D ). This passes the proximal end of the FCR tendon graft around the distal end as the graft passes from the index metacarpal base to the ulnar aspect of the thumb metacarpal base ( Fig. 2E ).

The end result of this weave is the passing of the FCR slip starting proximal and volar to the transverse ligament, dorsal to the base of the thumb metacarpal to the APL insertion, then back to the FCR insertion at the index metacarpal base, and then back to the APL insertion again ( Fig. 1E , Fig. 2F ). In lassoing the tendon graft in this fashion the portion of tendon graft that passes from the index metacarpal base to the ulnar aspect of the thumb metacarpal base replaces the function of the intermetacarpal ligament, and a portion of graft is proximal to the base of the thumb metacarpal functioning as a supportive sling.

The free end of tendon graft is then held in tension and sutured back to the APL where the graft has already been sutured ( Fig. 2G ). Lastly, imbrication of the APL tendon is performed, shortening it ∼1 cm and effecting abduction of the thumb metacarpal ( Fig. 2H , 3 )

**Fig. 3:**
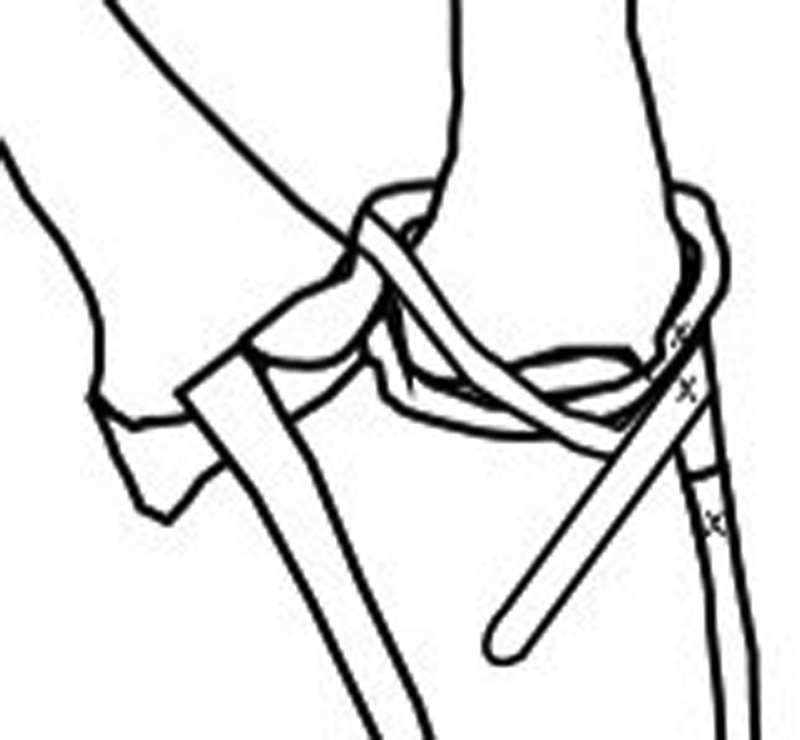
Schematic representation of completed ligament reconstruction.

The incision is then closed with absorbable sutures.

### Postoperative Care and Rehabilitation

Postoperatively, patients have the operated thumbs and wrists immobilized for 2 weeks using a thumb spica splint spanning the wrist and thumb. After their first follow-up appointment at 2 weeks, they are fitted with a removable thumb spica splint and permitted movements of the thumb under the guidance of a hand therapist.

## Results

Of the study group comprising all 86 patients meeting study criteria, the mean age at time of surgery was 62.8 years. Mean follow-up for this group was 13.1 months. Eighty-two were right handed and four left handed. Ten patients underwent surgery on both thumbs over the course of follow-up. Of those undergoing unilateral surgery, 41 of 76 (53.9%) underwent surgery on the dominant hand and the 35 on the nondominant hand.

Two patients underwent the surgery for revision of a failed prior arthroplasty procedure; both required removal of spacer implants (one silastic and the other metallic), while three patients had undergone prior carpal tunnel release.

Among the 66 patients with a minimum of 3 months of follow-up, 2 patients (3%) developed complications: one patient developed an early superficial infection that required treatment with oral antibiotics, and one patient had a recurrence of pain at the operative site and required periodic steroid injections for symptoms control.

With regard to functional outcomes, 65 patients were available for analysis of preoperative and postoperative grip strength dynamometry. Grip strength measurements in the operated hand had returned to the preoperative measurement by the 3 months mark: Preoperative: 40.7lbs ± 22.1, as compared with 3 months postoperative: 40.5lbs ± 20.0 ( *p* = 0.56).

Analysis of the nine patients who had dynamometry scoring more than 13 months from surgery showed a significant increase in grip strength in the operated hand from a mean of 40.7 lbs ± 22.1 to 54.7 lbs ± 24.1 ( *p* = 0.03). In these same nine patients, the grip strength measurements in both hands were also not significantly different from each other beyond 13 months.

Analysis of functional scores showed decreases in the following scores ( Table 1 ): DASH, DASH/QuickDASH9, Boston/Levine-Katz questionnaire symptom severity score (SSS), Boston/Levine-Katz questionnaire functional status scores. Reductions in the each of the scores listed indicate a reduction in disability, symptom severity, or functional impairment.

**Table 1:** Preoperative and postoperative functional score measurements

	Mean	SD	*p* -Value	*n*
Preoperative DASH	41.2	20.6	0.003	18
Postoperative DASH	24.8	21.4		
Preoperative Dash/QDASH9	43.9	21.6	<0.001	21
Postoperative Dash/QDASH9	25.4	19.9		
Preoperative Levine SSS	2.6	0.9	<0.001	22
Postoperative Levine SSS	1.7	0.5		
Preoperative Levine FSS	2.8	1.0	<0.001	22
Postoperative Levine FSS	1.8	0.7		

Abbreviations: DASH, disabilities of the arm shoulder and hand; FSS, functional severity score; QDASH9, QuickDASH9; SD, standard deviation; SSS, symptoms severity score.

## Discussion

Results of concurrent CTR via this approach with arthroplasty of the TMC joint have been described in the literature, but these have been limited to small series of not more than 10 patients. 15 16 In this single surgeon study, we report the favorable functional results that can be obtained with the senior author's technique of concurrent CTR and TMC arthroplasty with ligament reconstruction and a short period of immobilization. Studies and systematic reviews have failed to find superiority of any particular technique for the management of TMC OA, 7 8 and this may represent a ceiling effect; with the outcomes of most procedures being favorable, proving superiority of one technique over another is challenging. Among the various reconstruction techniques, no difference at 1 year was found between techniques involving bone tunnels and those not involving bone tunnels. The technique described above has several advantages: (1) the technique is straight forward and reproducible with the majority of the surgeries performed by hand surgery fellows under the direct supervision of the senior author, (2) it does not involve the drilling of bone tunnels thus obviating the risks associated with drilling bone tunnels, and (3) the period of immobilization for patients is short, with patients being splinted full time for 2 weeks and thereafter only requiring splinting intermittently.

Performing carpal tunnel release routinely at the time of TMC arthroplasty has been advocated by several authors 10 14 there are several reasons in favor of this approach. In our study, 14 of the 114 patients during the study period did not have a CTR performed at the same time as the TMC arthroplasty as these patients had undergone carpal tunnel release previously. Considering the high rate of concurrent CTS in patients with TMC OA, a high index of suspicion is required. Preoperative nerve conduction studies might aid in selecting patients for concurrent CTR; this, however, would still miss the percentage of patients with CTS and negative nerve conduction studies. It has also been shown that electrodiagnostic testing does not significantly change the probability of diagnosing CTS in patients diagnosed with CTS clinically. 17 The limited additional morbidity of CTR via the radial approach at the time of TMC arthroplasty described above requires no additional skin incision, minimal additional dissection, and no modification in postoperative care. The additional cost to the patient in direct spending and time off work if CTR is required later can also be avoided by performing CTR at the same sitting as TMC arthroplasty. These reasons underscore the approach of performing CTR routinely at the time of TMC arthroplasty in patients who have not undergone CTR previously.

Regarding functional measures, the minimal clinically important difference (MCID) in functional measures is of greater importance than just mere statistical significance. Methodologies for calculation of MCID vary, with several studies, 18 19 estimating it at 10 for the DASH with the upper boundary being 15 by the DASH web site. The decrease in mean DASH scores presurgery and postsurgery was 16.4 in this study demonstrating a change that is not just statistically significant, but one that is also clinically meaningful to patients. The MCID of SSS of the Boston questionnaire has also been investigated in particular in the setting of steroid injections for CTS. 20 In that study it was found to be 1.04. In our study, the difference in mean SSS was 0.9, and while approaching the MCID in that other study this may represent the relative dominance of the symptoms of arthritis over the patient's symptoms from the TMC OA.

There are several significant limitations to this study. It is a retrospective study of a small series, and there is no control group. Only 27% of the study population had both preoperative and postoperative functional scoring available for analysis, and only 14% of the study population had grip strength testing at more than 1 year of follow-up. Owing to the retrospective nature of the study, the number of patients whose medical records contained records of functional scoring and grip strength testing was limited. There was significant amount of missing data that precluded the inclusion of many patients for these portion of the analysis. Regarding the diagnosis of CTS, this was performed clinically but in an unstandardized fashion without the use of available standardized and validated diagnostic instruments (e.g., CTS-6 questionnaire 17 ). The overlap of symptoms between the CTS and TMC OA and the nature of the Boston/Levine-Katz questionnaire used for assessing symptoms of CTS limited our ability to distinguish the relief from symptoms from CTS from the relief from symptoms of TMC OA. The relief of overall symptoms, however, is captured well by the overall decrease in DASH scores. Lastly, there were few patients with long-term follow-up owing to the referral nature of the senior author's practice.

In conclusion, we present the favorable results of surgical and postoperative management of degenerative joint disease of the TMC joint and CTS using the senior author's unique technique of concurrent arthroplasty of the TMC joint and carpal tunnel release paired with a postoperative protocol involving short-term immobilization and early mobilization.

## Notes

**Erratum:** Dr. Tsu-Min Tsai's name and affiliation were corrected as per the erratum published online on November 21, 2019. The DOI of the erratum is 10.1055/s-0039-1700493.
