# Erratum: Trapeziometacarpal Joint Arthroplasty of the Thumb without Osseous Tunnels and Carpal Tunnel Release via a Radial Approach; Technique, and Results

**DOI:** 10.1055/s-0039-1700493

**Published:** 2019-11-21

**Authors:** Chung Ming Chan, Efraín Farías Cisneros, Tsu-Min Tsai

**Affiliations:** 1 Department of Orthopaedics and Rehabilitation, University of Florida, Gainesville, Florida; 2 Department of Orthopedics, Hospital Español de México, Mexico City, Mexico; 3 Christine M. Kleinert Institute for Hand and Microsurgery, Louisville, Kentucky

It has been brought to our attention that the name and affiliation of Dr. Tsu-Min Tsai appeared incorrectly in the above article published online in Volume 5, Issue 3 of *The Surgery Journal* (DOI: 10.1055/s-0039-1697635 ). The name and affiliations have now been corrected.

